# COVID-19 outcome trends by vaccination status in Canada, December 2020–January 2022

**DOI:** 10.14745/ccdr.v50i12a05

**Published:** 2024-01-01

**Authors:** Demy Dam, Sharifa Merali, Michelle Chen, Cameron Coulby, Brigitte Ho Mi Fane, Felix Bang, Jordan Robson, Samara David

**Affiliations:** 1 Centre for Emerging and Respiratory Infections and Pandemic Preparedness, Public Health Agency of Canada, Ottawa, ON; 2 Centre for Immunization Surveillance, Public Health Agency of Canada, Ottawa, ON

**Keywords:** COVID-19, vaccination, severe outcomes, surveillance, public health

## Abstract

**Background:**

The coronavirus disease 2019 (COVID-19) pandemic in Canada has evolved rapidly. Since late 2020, COVID-19 vaccines have been relied on to protect against severe outcomes in the presence of circulating variants of concern (VOC).

**Objective:**

This surveillance report provides a retrospective descriptive analysis of national trends in COVID-19 cases and severe outcomes by vaccination status, contextualizing trends against case demographics and circulating VOCs, from December 2020 to January 2022.

**Methods:**

Case and vaccination coverage surveillance data were obtained from the National COVID-19 Case Dataset and the Canadian COVID-19 Vaccination Coverage Surveillance System for 12 of 13 provinces and territories. Descriptive analyses were produced to describe trends over time among individuals aged 12 years and older by COVID-19 outcome, vaccination status, and demographics. Age-standardized and age-stratified incidence rates and incidence rate ratios were computed for cases, hospitalizations, and deaths.

**Results:**

From mid to late-2021, incidence rates for cases and severe outcomes were consistently lowest among those with a completed primary series and highest among those who were unvaccinated. Unvaccinated individuals were much more likely to be hospitalized or to die compared to those with a completed primary series in all variant periods. Age-specific rates of severe outcomes were consistently highest among those aged 80 years and older across all vaccination statuses.

**Conclusion:**

Vaccination remains one of the most important public health interventions, particularly among older adults, to protect against COVID-19 severe outcomes as the pandemic evolves. Routine monitoring of COVID-19 outcomes by vaccination status can identify changes in COVID-19 epidemiology and inform public health action and policy.

## Introduction

The coronavirus disease 2019 (COVID-19) pandemic has been one of the most significant public health crises in the last century, resulting in increased morbidity, mortality, and social and economic disruption in Canada and worldwide ((1,2)). Until vaccines were first authorized in Canada on December 9, 2020, broad and stringent public health measures (PHMs) were heavily relied on to slow transmission of the severe acute respiratory syndrome coronavirus 2 (SARS-CoV-2) and mitigate its impacts on health and society ((1,3)).

From December 2020 to January 2022, the COVID-19 pandemic in Canada evolved rapidly. Variants of the SARS-CoV-2 wild-type virus, including Alpha, Beta, Gamma, Delta, and Omicron, emerged, with differential impacts on COVID-19 outcomes ((3)). In Canada, public health falls under provincial/territorial jurisdiction and PHMs (e.g., travel restrictions, work/school closures, and personal protective measures, such as masking, and physical distancing) ((3)), testing strategies, and vaccine policies varied across provinces and territories (hereafter referred to as jurisdictions). Confirmatory COVID-19 testing was made broadly available across jurisdictions ((4)). Canadian COVID-19 vaccination campaigns began on December 14, 2020, initially prioritizing vulnerable and at-risk populations (3,5). Between March and November 2021, jurisdictions expanded vaccination eligibility from older adults to children older than five years, following changes to the availability of vaccines and recommendations from the National Advisory Committee on Immunization (NACI) ((5,6)). From September to December 2021, NACI recommended a booster dose of an authorized mRNA COVID-19 vaccine for key populations to address waning immunity and suboptimal primary series vaccine effectiveness ((7–9)). By January 1, 2022, 88% of people aged 12 years or older had received a primary COVID-19 vaccine series, and 19% had received a primary series with one additional dose (10).

This report provides a retrospective, descriptive analysis of national trends in COVID-19 outcomes by vaccination status among individuals aged 12 years and older, contextualized by variant and case demographic characteristics, from December 2020 to January 2022.

## Methods

### Data sources

Data were obtained from the National COVID-19 Case Dataset, a case-based surveillance system that collects data on demographics, clinical status and outcomes, risk factors, vaccination, and variant lineages of COVID-19 cases in Canada. Jurisdictions report case data electronically to the Public Health Agency of Canada (PHAC) at varying frequencies. Data are subsequently mapped and stored in a Postgres (PostgreSQL) database maintained by PHAC (Metabase).

Data on vaccination coverage (VC) estimates were obtained from provincial and territorial immunization repositories through the Canadian COVID-19 Vaccination Coverage Surveillance System (CCVCSS). The numbers of people vaccinated were aggregated by jurisdiction, week, age group (i.e., 12–17, 18–39, 40–59, 60–79, and 80+ years) and vaccination status. Yearly population estimates were obtained from Statistics Canada and supplemented by the Northwest Territories and Yukon governments. The unvaccinated population was calculated by subtracting the number of people with at least one dose of a COVID-19 vaccine from the population estimate. For the weeks when population estimates were lower than the population with at least one dose, the latter was used as the population estimate for each jurisdiction.

This analysis covers the period of December 14, 2020, the start of the Canadian COVID-19 vaccination campaign, to January 1, 2022. By January 1, 2022, most jurisdictions had reduced the scope of their testing strategies to prioritize individuals at higher risk of experiencing severe outcomes. For incidence rate analyses, the period of June 19, 2021, to January 1, 2022, was used due to VC data availability. Data were extracted on April 28, 2023, from the National COVID-19 Dataset and on April 23, 2023, from CCVCSS.

### Definitions

This analysis includes COVID-19 cases that met the national confirmed case definition ((11)). Vaccination statuses (defined in **Table 1**) were assigned using information about the number of doses received, time interval between vaccination and episode date, and vaccine product as per Health Canada authorization ((4)). Vaccination statuses were derived from VC definitions and include time to build immunity ((12)).

**Table 1:** Summary of vaccination status categories and definitions

Vaccination status	Definition
Unvaccinated	Cases with no recorded vaccine doses at time of the episode date.
Not yet protected	Cases whose episode date occurred less than 21 days after their first dose of the vaccine, as per NACI dose interval recommendations ((2)).
Partially vaccinated	Only applies to two-dose series vaccines. Cases whose episode date was 21 days or more after receipt of first vaccine dose or less than 14 days after receipt of second vaccine dose of a Health Canada authorized vaccine.
Primary series completed	Cases whose episode date was 14 days or more after receipt of a second dose in two-dose series, 14 days or more after receipt of one dose of a one-dose vaccine, or 0 to <14 days after receipt of a first additional dose (e.g., third or booster) of a Health Canada authorized COVID-19 vaccine.
Primary series completed with one additional dose^a^	Cases whose episode date was 14 days or more following the receipt of one additional dose of a Health Canada authorized vaccine, after completing a primary series. Individuals who received one additional dose prior to September 28, 2021 (e.g., as part of a three-dose primary series or for travel purposes), were categorized as primary series completed.
Unknown status	• Cases with missing or “unknown” value in the vaccinated variable (yes/no). • Cases with missing or “unknown” vaccine product names. • Cases with approved vaccine but the respective vaccination date is missing. • Cases with vaccine products not authorized by Health Canada. • Cases with vaccination dates before December 14, 2020. • Cases with a second dose of primary series administered less than 21 days after the receipt of a first dose. • Cases with vaccination dates for booster doses less than 14 days after the previous vaccine dose. • Cases with the Medicago Covifenz vaccine product. Approval for this product was given in 2022 and later discontinued by Health Canada as of April 17, 2023 ((13)). • Cases with vaccine product of COVISHIELD received after Health Canada authorization had expired on September 16, 2021 ((14)).

Abbreviations: COVID-19, coronavirus disease 2019; NACI, National Advisory Committee on Immunization

^a^ Based on NACI recommendations for additional doses ((8)), this vaccination status group was incorporated into analyses as of September 28, 2021

### Data analysis

Descriptive statistics were computed to explore demographic and clinical characteristics of COVID-19 cases by vaccination status. To visualize changes in VC in Canada, the proportion of the population with a completed primary series was plotted over time by age group and a 14-day lag was applied to coverage counts to account for time to build immunity ((12,15)).

To contextualize changes in severity and transmissibility due to circulating variants, six variant periods were defined: wild-type, mixed variant of concern (VOC) emergence, mixed VOC predominance, Delta emergence, Delta predominance, and Omicron emergence in Canada. The start and end of a variant predominance period occurred when the specified variant first and last accounted for 75% of sequenced cases, hospitalizations, ICU admissions, and deaths. When cases, hospitalizations, ICU admissions, or deaths predominance dates were different, the latest start date was used to capture the most specific cut point for all indicators. Emergence periods were defined as the day following the end of a predominance period until the next variant reached predominance. The mixed VOC period includes the Alpha, Beta, and Gamma variants, as no single VOC represented over 75% of sequenced cases.

Incidence rates were calculated using VC data as denominators. Denominator data were not available during this analysis period for cases with a complete primary series and one additional dose; these cases were grouped with those with a complete primary series for incidence rate calculations. Population estimates were used to calculate population fractions by age group for computing weekly age-standardized incidence rates for cases, hospitalizations, and deaths. To compare trends between variant periods, the average weekly incidence rate and incidence rate ratios (IRR) of these averages were computed by vaccination status for each variant period, similar to previously published methodologies using COVID-19 surveillance data ((16,17)). Case data were cleaned using SQL in Metabase and were analyzed using R Statistical Software version 4.0.4.

### Data quality, missing data, and reporting delays

Vaccination data were available for 12 of 13 jurisdictions (all except Québec), representing 78% of the Canadian population (18). Cases less than 12 years old were excluded since they were not eligible for vaccination until November 19, 2021 ((6)). Cases with missing information on vaccination status or age were excluded. Cases categorized as “not yet protected,” “primary series completed and two additional doses,” and “unknown status” were excluded. Vaccination coverage was consistently reported by jurisdictions beginning June 5, 2021; as such, incidence rates were calculated for cases with episode dates of June 19, 2021, onward (accounting for two weeks to build immunity) ((19)).

## Results

**Figure 1:**
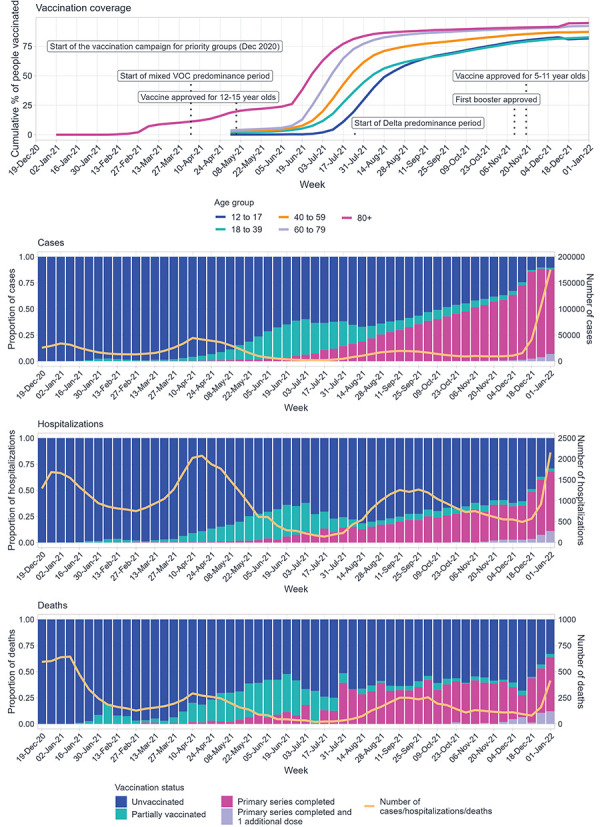
Vaccination coverage of the Canadian population^a^, December 14, 2020, to January 1, 2022

Vaccination coverage gradually increased in Canada from December 2020 as vaccination eligibility expanded, with the proportion of individuals aged 12 years and older with a completed primary series reaching over 80% by the end of 2021 (**Figure 1**). From late-2020 to mid-2021, unvaccinated individuals accounted for the highest proportion of cases; however, this proportion decreased as more vaccinated individuals became cases in late 2021, aligning with the gradual increase in VC. A larger increase in the proportion of vaccinated cases was noted in December 2021, following the emergence of the Omicron variant ((20)).

From December 14, 2020, to January 1, 2022, a total of 1,194,694 COVID-19 cases with complete case-level vaccination history (73.6% of all cases) over the age of 12 years were reported to PHAC (**Table 2**). The majority of these cases were unvaccinated, and the lowest proportion of cases was among those with a completed primary series and one additional dose. The proportion of cases was higher among females than males. Unvaccinated cases were generally younger than vaccinated cases. The highest proportions of hospitalized cases and deaths were reported among those who were unvaccinated, followed by those who completed a primary series.

**Table 2:** Descriptive statistics of cases by vaccination status and demographics and outcomes, December 14, 2020, to January 1, 2022

Demographic characteristic or outcome	Unvaccinated (N=748,456)	Partially vaccinated (N=57,995)	Primary series completed (N=370,574)	Primary series completed and one additional dose (N=17,669)	Overall^a^ (N=1,194,694)
Female	364,036 (48.8%)	30,460 (52.6%)	198,372 (53.7%)	11,232 (63.7%)	604,100 (50.7%)
Male	382,161 (51.2%)	27,457 (47.4%)	170,969 (46.3%)	6,390 (36.3%)	586,977 (49.3%)
12–17 years	66,535 (8.9%)	2,824 (4.9%)	22,598 (6.1%)	36 (0.2%)	91,993 (7.7%)
18–39 years	366,261 (48.9%)	23,340 (40.2%)	178,406 (48.1%)	5,142 (29.1%)	573,149 (48.0%)
40–59 years	217,499 (29.1%)	16,262 (28.0%)	117,101 (31.6%)	6,139 (34.7%)	357,001 (29.9%)
60–79 years	80,988 (10.8%)	11,895 (20.5%)	43,190 (11.7%)	4,752 (26.9%)	140,825 (11.8%)
80+ years	17,173 (2.3%)	3,674 (6.3%)	9,279 (2.5%)	1,600 (9.1%)	31,726 (2.7%)
Cases hospitalized	42,708 (80.9%)	3,592 (6.8%)	6,116 (11.6%)	394 (0.7%)	52,810 (100.0%)
Cases deceased	8,309 (78.3%)	767 (7.2%)	1,450 (13.7%)	84 (0.8%)	10,610 (100.0%)

^a^ Cases with vaccination status of “Not yet protected” were excluded from this analysis

**Figure 2:**
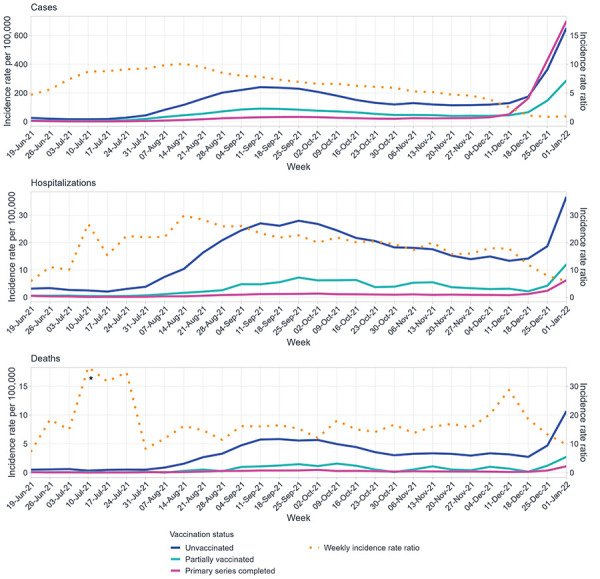
Weekly age-standardized incidence rate of COVID-19 cases, hospitalizations, and deaths per 100,000 population by vaccination status, June 19, 2021, to January 1, 2022^a,b^ ^a^ Weekly incidence rate ratios compare those unvaccinated to those with a completed primary series ^b^ Incidence rate for primary series completed was zero during the week of July 10, 2021, as no deaths were reported

During the Delta emergence period, age-standardized incidence rates for all COVID-19 outcomes remained low for all vaccination statuses (**Figure 2** and **Table 3**). During the Delta predominance period, incidence rates increased for cases in August; severe outcomes peaked in mid-September, remaining elevated until mid-December. The increase was more pronounced among unvaccinated cases. Incidence rates for cases, hospitalizations, and deaths were consistently highest in the unvaccinated and lowest in those with a completed primary series from mid to late-2021. However, in mid-December 2021, overall cases and severe outcomes rapidly increased, and the case incidence rate among those who completed a primary series surpassed that of the unvaccinated.

**Table 3:** Average of the weekly incidence rates and incidence rate ratios for cases, hospitalizations, and deaths by vaccination status and variant period, June 19, 2021^a^ to January 1, 2022

Variant period (date range)	Cases and severe outcomes	Average weekly incidence rates (per 100,000 population)			Average weekly incidence rate ratio
		Unvaccinated	Partially vaccinated	Primary series completed	Unvaccinated/primary series completed
Delta emergence (May 31, 2021^a^–July 24, 2021)	Cases	21.4	6.7	3.1	6.8
	Hospitalizations	2.8	0.5	0.2	11.4
	Deaths	0.5	0.08	0.03	17.5
Delta predominance (July 25, 2021–December 5, 2021)	Cases	152.6	57.3	24.7	6.2
	Hospitalizations	18.4	4.0	0.8	21.0
	Deaths	3.6	0.7	0.2	15.4
Omicron emergence (December 6, 2021–January 1, 2022)	Cases	396.5	166.7	430.2	0.9
	Hospitalizations	23.2	6.1	3.3	7.1
	Deaths	6.0	1.4	0.5	11.3

^a^ Although the Delta emergence period began on May 31, 2021, vaccination coverage denominator data for incidence rate calculations were not available until June 19, 2021

**Figure 3:**
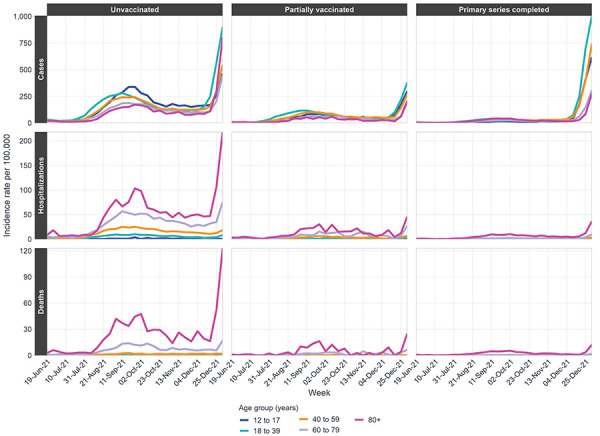
Weekly age-stratified incidence rate of COVID-19 cases, hospitalizations, and deaths per 100,000 population by 20-year age group and vaccination status, June 19, 2021, to January 1, 2022

Incidence rate ratios (IRR) for unvaccinated cases, hospitalizations, and deaths compared to those with a completed primary series remained high for most of the year (Figure 2). During the Delta emergence period, unvaccinated people were 11.4 and 17.5 times as likely to be hospitalized or to die due to COVID-19, respectively, compared to people who completed a primary series (Table 3). During the Delta predominance period, the IRR of the unvaccinated compared to those with a completed primary series increased for hospitalizations and decreased for deaths, compared to the Delta emergence period. During the Omicron emergence period, there was a decrease in IRR for cases, hospitalizations, and deaths, when compared to the Delta predominance period, though the IRR for cases had a more pronounced decrease. Following the emergence of the Omicron variant in mid-November, the weekly IRR decreased as more vaccinated people became infected (**Figure 3**).

Age-specific rates of severe outcomes were consistently highest among those aged 80 years and older, followed by those aged 60 to 79 years, for all vaccination statuses (Figure 3). Case incidence rates were highest among individuals aged 18 to 39 years, followed by those aged 40 to 59 years, from mid-2021 to late August 2021. The incidence rate of cases in these two age groups declined following VC increases until the Omicron emergence period.

## Discussion

### Summary of key results

From December 14, 2020, to January 1, 2022, a total of 1,194,694 cases of COVID-19 over 12 years of age and with complete case-level vaccination history were reported to PHAC. From mid to late-2021, incidence rates for cases, hospitalizations, and deaths were consistently highest among unvaccinated people and lowest among those with a completed primary series. In December 2021, following the emergence of the Omicron variant, the case incidence rate among those who completed a primary series surpassed that of the unvaccinated; however, rates of severe outcomes remained lower among those who completed a primary series.

### Key results and comparison

#### Vaccination coverage and case incidence

Vaccination coverage steadily increased in Canada as eligibility expanded, varying by age groups ((3,12)). Like in the United Kingdom, case incidence in 2021 was consistently highest in unvaccinated individuals, with younger age groups having the highest incidence rates ((21,17)). Starting in spring 2021, there was an increase in vaccine breakthrough cases, consistent with studies showing that, although completion of a primary vaccination series was highly effective in preventing infection against the wild-type virus and Alpha variant, it was slightly less effective against the Beta, Gamma, and Delta variants ((22)).

#### Severe outcomes

Incidence rate ratio analyses showed that people who were unvaccinated were much more likely to be hospitalized and to die from COVID-19 than those who completed a primary series during the Delta emergence, Delta predominance, and Omicron emergence periods. Although IRRs for hospitalization and death decreased during the Omicron emergence period, protective effects against severe outcomes were still observed in those who completed a primary series. These trends are similar to those reported in the United States during the same period ((17)). Incidence rates for severe outcomes were highest in individuals aged 80 years and older, in agreement with studies showing that advanced age increased the risk of COVID-19 death ((23,16)). Among individuals aged 80 years and older, severe outcomes were lowest in those who completed their primary series, followed by those who were partially vaccinated, consistent with studies illustrating that a primary vaccine series was highly protective against severe outcomes from the Alpha, Beta, Gamma, Delta, and Omicron variants ((16,17,21)), and that partial vaccination was also effective at preventing hospitalizations and deaths ((15,24)).

#### Waning immunity during Delta period

When the more severe and transmissible Delta variant was predominant ((25)), there was an increase in the incidence of cases among individuals who completed a primary series, similar to trends observed in other countries ((17,26–28)). This shift could be due to potential waning of vaccine-induced immunity against symptomatic infection ((29,30)), a longer time since vaccination ((26,27)), and reduced effectiveness of available vaccines at preventing infection against the Delta variant (22). Although case IRRs decreased during the Delta predominance period, IRRs for hospitalizations and deaths remained high, suggesting that a primary series was still protective against severe outcomes, consistent with the literature ((17,26)). Age-stratified incidence rates for hospitalizations and deaths in fall 2021 were substantially higher among cases aged 80 years and older across all vaccination statuses. This age group was prioritized for vaccination and completed their primary series earlier in the year, further raising concerns about waning immunity (26). Routine monitoring of severe outcomes following vaccination helped inform NACI booster dose recommendations, which recommended earlier booster shots for individuals at higher risk of severe illness ((8)).

#### Emergence of the Omicron variant

The introduction of the immuno-evasive Omicron variant ((20)) in mid-November 2021 was followed by a resurgence in cases and severe outcomes, corresponding to when over 85% of the population over the age of 12 years had completed their primary series. Although there was a substantial increase in case incidence among individuals who completed a primary series, the increases in the incidence of hospitalizations and deaths were proportionally lower than that of cases. Moreover, even with the increase in case incidence following this case resurgence, severe outcomes remained lowest among those who were vaccinated. Primary vaccination series still conferred good protection against severe outcomes from the Omicron variant, despite reduced protection against infection (17,22). Studies showed that a booster dose offered additional protection against infection and severe outcomes with the Omicron variant ((23,31–33)).

## Strengths and limitations

Of 13 jurisdictions in Canada, 12 and 13 regularly reported case-level vaccination history data and VC data to PHAC, respectively. Strong participation and collaboration between federal and jurisdictional entities in Canada, alongside widespread community testing, enabled monitoring of highly representative national trends in COVID-19 outcomes following vaccination during this period. As such, the differential impacts of vaccination on COVID-19 outcomes by demographics and SARS-CoV-2 variants were effectively captured as vaccines became more widely available and administered in Canada.

Vaccination rollout timelines and uptake, testing strategies, PHMs, and VOC emergence differed across and within jurisdictions; therefore, national trends should be interpreted with caution. Denominator data were not available before June 2021 as VC data was not consistently reported from jurisdictions, precluding analysis of more stable incidence rate trends during this period. Public health testing in many jurisdictions prioritized high-risk individuals and healthcare workers during this period, which may have introduced bias into earlier descriptive trends. Additionally, distribution of rapid antigen tests to the population could lead to an underestimate of PCR-confirmed cases in late 2021. The overlap of variant periods with circulating VOCs could have introduced bias in the results. The analysis period ends shortly after the emergence of the Omicron variant and does not fully capture waning of vaccine-induced immunity. Analyses could not account for reinfection and natural or hybrid immunity, as these data were not available. This analysis does not include cases aged younger than 12 years, as they were not eligible for vaccination for most of the analysis period. Demographic data were limited to age and gender, as data on race/ethnicity and socioeconomic status were not available. Lastly, cases that were excluded from the analysis due to missing or unknown data (e.g., vaccination status) may differ from those that were included by characteristics for which data were not available (e.g., health conditions).

## Conclusion

In Canada, hospitalizations and deaths due to COVID-19 were highest in older age groups; however, vaccination reduced the incidence of severe outcomes by a notable margin across all age groups. Routine monitoring of COVID-19 outcomes by vaccination status is an important pillar in Canadian COVID-19 epidemiology and surveillance to understand the impact of vaccines across Canada. It has informed Canadians about the COVID-19 epidemiologic situation in Canada and provided evidence to support policies, directives, and recommendations on vaccination and public health interventions from NACI, the PHAC Office of the Chief Public Health Officer, and jurisdictions. Though the landscape of COVID-19 is ever-changing, vaccination remains one of the most important public health interventions to protect against COVID-19 severe outcomes.
